# Evaluation of the efficacy, safety and tolerability of orally administered BI 409306, a novel phosphodiesterase type 9 inhibitor, in two randomised controlled phase II studies in patients with prodromal and mild Alzheimer’s disease

**DOI:** 10.1186/s13195-019-0467-2

**Published:** 2019-02-12

**Authors:** Lutz Frölich, Glen Wunderlich, Claus Thamer, Michael Roehrle, Miguel Garcia, Bruno Dubois

**Affiliations:** 1 Department of Geriatric Psychiatry, Central Institute of Mental Health, Mannheim, Germany; 2 Boehringer Ingelheim (Canada) Ltd., Burlington, ON Canada; 3 Boehringer Ingelheim International GmbH. KG, Biberach an der Riss, Germany; 4 Boehringer Ingelheim Pharmaceuticals Inc., Ridgefield, CT USA; 5 Institut de la Mémoire et de la Maladie d’Alzheimer (IM2A), Centre des Maladies Cognitives et Comportementales, Hôpital La Salpêtrière, Paris, France

**Keywords:** Alzheimer’s disease, Treatment efficacy, Neuropsychological test battery, Phosphodiesterase type 9 inhibitor, Prodromal stage, Safety

## Abstract

**Background:**

There are currently no approved treatments for the prodromal stage of Alzheimer’s disease (AD). Approved symptomatic treatments for mild-to-moderate AD include acetylcholinesterase inhibitors and memantine, but more efficacious treatments are needed. BI 409306 is a potent and selective phosphodiesterase 9 inhibitor assessed for the symptomatic treatment of AD. Efficacy and safety of BI 409306 was analysed in two phase II proof-of-concept clinical trials in cognitive impairment associated with prodromal AD (study 1) and mild AD (study 2).

**Methods:**

Two multicentre, double-blind, parallel-group, randomised controlled phase II studies were conducted (North America/Europe). Following study run-in, eligible subjects were randomised to one of four oral doses of BI 409306 (10–50 mg daily) or placebo (1:1:1:1:2 ratio) for 12 weeks. The primary efficacy endpoint was the change from baseline in Neuropsychological Test Battery (NTB) total *z*-score after 12 weeks’ treatment. Secondary efficacy assessments included change from baseline in Clinical Dementia Rating scale-Sum of Boxes (CDR-SB), Alzheimer’s Disease Assessment Scale-cognitive subscale (ADAS-Cog11) and Alzheimer’s Disease Cooperative Study-Activities of Daily Living scale (ADCS-ADL; mild cognitive impairment [MCI] version for prodromal patients) after 12 weeks’ treatment. Safety and tolerability assessments included adverse event reporting and vital sign monitoring. Change from baseline in NTB total *z*-score (primary endpoint) and CDR-SB were analysed using the restricted maximum likelihood-based mixed-effects model with repeated measurement. An analysis of covariance model was used to assess other secondary endpoints.

**Results:**

Four hundred fifty-seven patients were randomised (study 1 for prodromal AD, *N* = 128; study 2 for mild AD, *N* = 329); 427 (93.4%) completed.

A prespecified pooled analysis of the primary endpoint revealed no significant changes in NTB total composite *z*-score at week 12 in the BI 409306 treatment groups compared with placebo, with similar findings observed in the individual studies. The analysis of all secondary endpoints, including pooled analysis of CDR-SB and ADAS-Cog11, ADCS-MCI-ADL (study 1), ADCS-ADL (study 2), also gave no indication of a treatment benefit for BI 409306, compared with placebo. BI 409306 was well tolerated.

**Conclusions:**

Overall, the data do not demonstrate efficacy of BI 409306 in improving cognition in patients with prodromal and mild AD. BI 409306 is well tolerated.

**Trial registration:**

ClinicalTrials.gov, NCT02240693 and NCT02337907. Registered 15 September 2014 and 09 January 2015, respectively.

## Background

Alzheimer’s disease (AD) is a neurodegenerative brain disorder and the most common cause of dementia, accounting for up to 70% of dementias occurring in older adults [1, 2]. Prodromal AD corresponds to the early pre-dementia stage of AD, characterised by a noticeable and measurable decline in cognitive abilities, including impairment of episodic memory and in vivo structural and biologic evidence of AD pathology [3]. There are currently no approved treatments for the prodromal stage of AD; however, providing a treatment option for patients at this stage of disease would allow for a targeted approach in addressing early cognitive deficits, which could delay the onset of more severe symptoms.

**Fig. 1:**
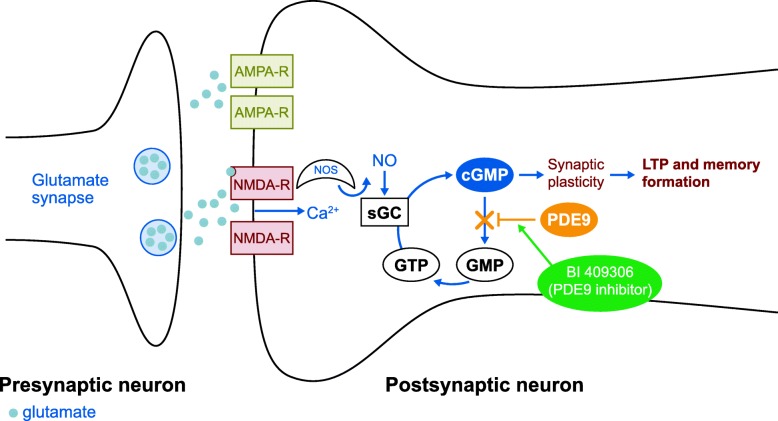
Putative mode of action of phosphodiesterase type 9 inhibition by BI 409306. Ca^2+^, calcium; cGMP, cyclic guanosine monophosphate; GTP, guanosine triphosphate; LTP, long-term potentiation; NMDA-R, *N*-methyl-D-aspartate receptor; NO, nitric oxide; NOS, nitric oxide synthase; PDE9, phosphodiesterase type 9; sGC, soluble guanylate cyclase. Figure adapted from Moschetti et al. [7]

One of the key characteristics of AD is an abnormality in glutamatergic neurotransmission related to *N*-methyl-d-aspartate (NMDA) function in the cortical and hippocampal regions of the brain [4]. Activation of the NMDA receptor signalling pathway produces post-synaptic signalling events via elevation of secondary messengers, such as cyclic guanosine monophosphate (cGMP) [5]. In conditions of NMDA receptor hypofunction, such as AD, it is hypothesised that inhibition of phosphodiesterase type 9 (PDE9), which hydrolyses cGMP [6], may increase cGMP levels and improve NMDA receptor signalling (Fig. 1 [7]). This would lead to strengthened synaptic plasticity and stabilisation, via enhanced long-term potentiation (LTP), therefore potentially improving cognitive functions [5, 7–9].

BI 409306 is a PDE9 inhibitor that has been shown to enhance LTP and hypothesised to improve cognitive function and memory by strengthening synaptic plasticity [7]. BI 409306 has been shown to potently and selectively inhibit PDE9 in rodents, resulting in a dose-dependent increase in cGMP levels in the prefrontal cortex and cerebrospinal fluid (CSF) [10–13]. In addition, results from a first-in-human, single-dose (≤ 350 mg) trial of BI 409306 showed that the absorption and elimination of BI 409306 was rapid, and plasma concentrations increased with dosage. BI 409306 was also shown to be generally well tolerated in healthy male subjects [7].

Following on from these findings, the primary objective of the studies described was to assess the efficacy and safety of BI 409306 at doses of 10–50 mg daily over a 12-week treatment period in two phase II proof-of-concept studies in prodromal and mild AD.

## Methods

### Study design

Two multicentre, double-blind, parallel-group, randomised controlled phase II studies were conducted in 12 countries to investigate the efficacy, safety and tolerability of orally administered BI 409306 during a 12-week treatment period compared with placebo, in patients with AD. Study 1 (NCT02240693) included patients with cognitive impairment associated with prodromal AD (Mini-Mental State Examination [MMSE] ≥ 24 and Clinical Dementia Rating [CDR] total score 0 or 0.5) diagnosed according to International Working Group (IWG) criteria [14]. Study 2 (NCT02337907) included patients with mild AD (MMSE 18–26 and CDR total score ≥ 1) according to National Institute on Aging-Alzheimer’s Association (NIA-AA) recommendations [15].

A prespecified pooled analysis of both studies was performed. This decision was based on (i) the expectation that the mechanism of action of BI 409306 would make it efficacious in the treatment of both prodromal and mild AD, (ii) the similarity of study designs and (iii) to overcome any possible operational difficulties in recruitment.

After obtaining informed consent, patients underwent a screening period of 5 weeks or 1 week in studies 1 and 2, respectively. A longer screening period was required for study 1 to accommodate testing for biomarkers associated with prodromal AD and, where necessary, brain imaging to rule out cerebrovascular causes of mild cognitive impairment (MCI). The screening period was followed by a 2–3-week, single-blinded, placebo run-in period before randomisation.

**Fig. 2:**
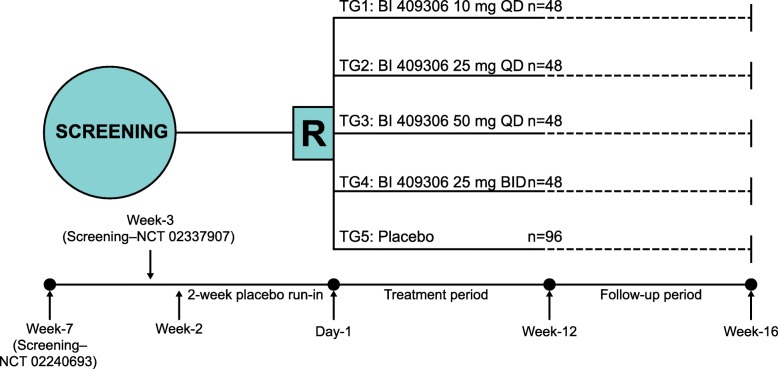
Study design for prodromal and mild Alzheimer’s disease studies. All *n* numbers represent the planned values. BID, twice daily, QD, once daily; R, randomisation

During the run-in period, placebo tablets were administered daily (two in the morning and one in the evening) to mimic the BI 409306 dosing schedule during the treatment period. Using Interactive Response Technology, patients were then randomised 1:1:1:1:2 to one of five treatment arms (10, 25 or 50 mg once daily [QD] or 25 mg twice daily [BID] BI 409306 or placebo per os) (Fig. 2). For blinding reasons, all treatments consisted of two tablets of active drug or placebo for the morning dose followed by one tablet of active drug or placebo for the evening dose, depending on treatment arm. BI 409306 and placebo tablets were identical in size and appearance. A follow-up visit took place at 28 days after the end of the 12-week treatment period.

Originally, an additional treatment arm of donepezil (an active comparator) 10 mg QD (starting with 5 mg for the first 4 weeks) was included in study 2 (*n* = 5); however, this arm was removed early from the study via a protocol amendment, which allowed enrolment of patients taking concomitant acetylcholinesterase inhibitors (AChEIs), as permitted in the inclusion criteria.

### Patients

Subjects were aged ≥ 55 years (with those aged > 85 years included, based on acceptable general health status) with body mass of ≥ 50 kg, an MMSE score of ≥ 24 (study 1) or 18–26 (study 2) and a CDR total score of 0 or 0.5 (study 1) or ≥ 1 (study 2), and were of non-childbearing potential or using acceptable birth control methods (if female) or were abstinent or using effective contraception (if male). Previous use of AD medications (AChEIs and/or memantine) was permitted up to 3 months prior to screening and, in study 2 only, current AChEI use was allowed if on a stable dose for ≥ 3 months prior to screening.

In study 1 only, subjects also had confirmed abnormal markers of AD pathology via either (i) presence in CSF of low amyloid beta peptide 1–42 (Aβ1–42) concentrations (< 640 pg/mL) and increased total tau concentrations (> 375 pg/mL) and/or low Aβ1–42 concentrations (< 640 pg/mL) and increased phospho-tau concentrations (> 52 pg/mL), or (ii) abnormal amyloid deposition in a cerebral positron emission tomography scan, and had episodic memory dysfunction assessed by the Free and Cued Selective Reminding Test (free recall test score ≤ 20 [of 48] and total recall test score ≤ 42 [of 48]) or Wechsler Memory Visual Paired Associates test (cognitive deficit of worse than 1 standard deviation [SD] to the mean, compared with the reference values of age and educational norms for inclusion). In study 2 only, subjects had an Alzheimer’s Disease Assessment Scale-cognitive subscale (ADAS-Cog11) score > 12.

Main exclusion criteria were substantial concomitant cerebrovascular disease, medical history of cancer within the last 5 years, history of symptomatic and unstable/uncontrolled conditions (based on investigator’s judgement) or of drug dependence or abuse, unstable/uncontrolled major depression, renal impairment, suicidal behaviour in the past 2 years, human immunodeficiency virus infection, previous participation in investigational drug studies of MCI or Alzheimer’s-type dementia within 3 months prior to screening and initiation of certain treatments within 3 months prior to randomisation (including tricyclic antidepressants, monoamine oxidase inhibitors, neuroleptics [with moderate/greater anticholinergic potency] and anticholinergic medications). Subjects were also excluded if dementia was secondary to another disorder (study 2 only).

Both studies were approved by their respective institutional review board/independent ethics committee and the competent authorities as per national and international regulations and conducted in accordance with the International Council for Harmonisation (ICH) of Technical Requirements for Pharmaceuticals for Human Use Guideline for Good Clinical Practice (GCP) and local legislation, as per the principles of the Declaration of Helsinki [16, 17]. All patients provided signed and dated written informed consent prior to study procedures in accordance with ICH GCP guidelines and the regulatory and legal requirements of the participating country.

### Study endpoints

#### Efficacy endpoints

The primary efficacy endpoint for both studies was change from baseline in Neuropsychological Test Battery (NTB) total *z*-score after the 12-week treatment period. Secondary endpoints, measured in both studies at the end of the 12-week treatment period, were the change from baseline in Clinical Dementia Rating scale-Sum of Boxes (CDR-SB) and the change from baseline in ADAS-Cog11. Additional secondary endpoints were the change from baseline in the AD Cooperative Study-Activities of Daily Living scale (ADCS-ADL) for MCI (ADCS-MCI-ADL) in study 1 and the ADCS-ADL in study 2. An additional secondary endpoint, assessed at the end of the 12-week treatment period, was the change from baseline in NTB subscales (memory, executive-function and immediate-memory domains).

#### Safety endpoints

Safety assessments included occurrence of adverse events (AEs) and serious AEs (SAEs), specified AEs of special interest indicative of drug-induced liver injury, laboratory parameters, physical and neurological examination, vital signs, electrocardiography and suicidality as judged by the Columbia-Suicide Severity Rating Scale.

#### Data quality assurance

A robust data quality assurance programme was employed, coordinated centrally with the services of an external vendor, to help ensure the quality and integrity of the efficacy data. The services included rater prequalification and central rater training for the neuropsychological assessments used as primary and secondary endpoints (online and at investigator meetings), provision of rater materials and central quality review of assessments including review of audio recordings of assessments.

### Statistical analysis

The primary endpoint, NTB total score, included nine tests. The mean NTB response of all pooled BI 409306 doses, versus the mean NTB response of placebo, was assessed using the following primary analysis model. For each patient, raw scores were converted to a standardised *z*-score using baseline means and SDs for each test (calculated using all randomised patients). Total *z*-score was obtained from an average of all resulting *z*-scores, and changes from baseline were therefore calculated as post-baseline composite *z*-score minus pre-treatment *z*-score, whereby a positive change indicated an improvement from baseline [18]. A restricted maximum likelihood (REML)-based mixed-model repeated measurement was used to assess the change from baseline in NTB total *z*-score after the 12-week treatment period. The model included fixed, categorical effects of treatment, visit, current AChEI use (yes/no; study 2 only), prodromal versus mild AD (pooled analysis only) and treatment by visit interaction, as well as the continuous fixed covariates of baseline and baseline-by-visit interaction. Unstructured covariance was used to model within-patient errors, and the Kenward–Roger approximation was used to estimate denominator degrees of freedom. If more than three out of nine NTB items were missing, the total *z*-score was not derived and was set to missing. Completely missing visits were handled through the statistical model.

Analysis of covariance was performed for the ADCS-MCI-ADL/ADCS-ADL and ADAS-Cog11 secondary efficacy endpoints. The REML-based mixed-effects model was used to analyse the CDR-SB endpoint, and the NTB subscale analyses (additional efficacy endpoint) used the same model as the primary endpoint. No multiplicity adjustments were made; hence, all *p* values are descriptive. All tests were done at a two-sided alpha level of 0.05.

#### Study populations

In total, 288 eligible patients were planned for inclusion in each of the individual studies, comprising an individual study sample size of 48 per active treatment arm and 96 per placebo arm, with statistical power to detect an effect size of 0.5 with 80% power at a two-sided alpha significance level of 5%. Based on the combined studies’ sample size, statistical power to detect an effect size of 0.43 with 80% power was achieved for individual pairwise comparisons. The sample size of the combined studies provided the ability to detect an effect size of 0.31 at 80% power for pooled BI doses versus placebo. The full analysis set, used for the primary analyses, included all randomised patients who were treated with ≥ 1 dose of study drug and had baseline and ≥ 1 post-baseline measurements for the primary endpoint. The treated set, used for safety analyses, included all patients who were randomised and treated with ≥ 1 dose of BI 409306.

## Results

### Study population and patient disposition

**Fig. 3:**
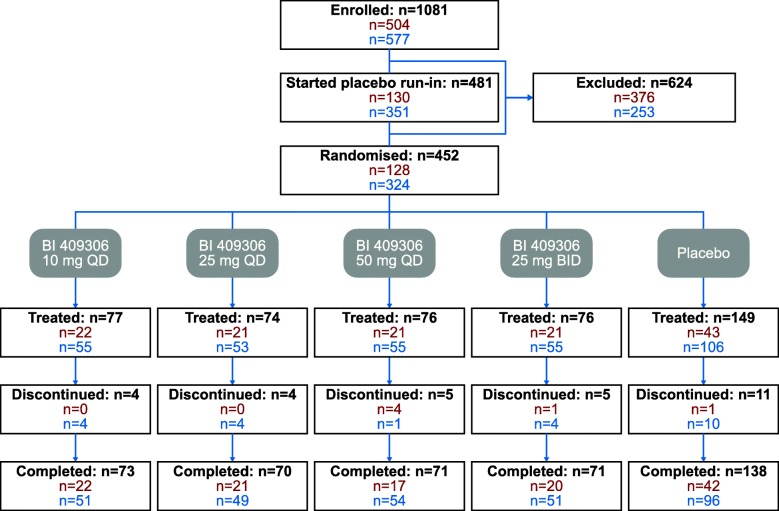
Disposition of patients. *BID* twice daily, *QD* once daily

Overall, 457 patients were randomised across both studies (*N* = 128 and *N* = 329 for study 1 and 2, respectively), with 427 (93.4%) completing them. Patient disposition across both studies is shown in Fig. 3, and patient demographics and baseline characteristics are described in Table 1. Baseline characteristics were similar between individual studies. Patients in the pooled analysis had a mean age of 73.6 years, 48.8% were male and the majority (98.9%) were White. The mean (SD) scores for the MMSE, CDR and ADAS-Cog11 for patients in the pooled analysis were 23.0 (3.3), 0.92 (0.37) and 18.31 (8.18), respectively.

**Table 1:** Patient demographics and baseline characteristics (pooled analysis)

	BI 409306 10 mg QD (*n* = 77)	BI 409306 25 mg QD (*n* = 74)	BI 409306 50 mg QD (*n* = 76)	BI 409306 25 mg BID (*n* = 76)	Placebo (*n* = 149)
Male, *n* (%)	41 (53.2)	32 (43.2)	42 (55.3)	33 (43.4)	73 (49.0)
Age (years), mean (SD)	73.3 (7.7)	74.2 (7.8)	73.1 (6.1)	74.0 (8.4)	73.5 (7.4)
Race, *n* (%)					
Asian	1 (1.3)	0 (0)	1 (1.3)	0 (0)	0 (0)
Black or African American	0 (0)	0 (0)	0 (0)	0 (0)	3 (2.0)
White	76 (98.7)	74 (100.0)	75 (98.7)	76 (100.0)	146 (98.0)
Body mass index, (kg/m^2^), mean (SD)	26.0 (3.4)	27.2 (5.2)	26.7 (3.7)	25.5 (3.9)	26.2 (4.1)
Smoking status, *n* (%)					
Never smoked	38 (49.4)	47 (63.5)	43 (56.6)	44 (57.9)	79 (53.0)
Ex-smoker	30 (39.0)	24 (32.4)	30 (39.5)	25 (32.9)	61 (40.9)
Current smoker	9 (11.7)	3 (4.1)	3 (3.9)	7 (9.2)	9 (6.0)
Alcohol status, *n* (%)					
Non-drinker	36 (46.8)	30 (40.5)	29 (38.2)	37 (48.7)	72 (48.3)
Drinks—no interference	41 (53.2)	44 (59.5)	47 (61.8)	39 (51.3)	77 (51.7)
Drinks—possible interference	0 (0)	0 (0)	0 (0)	0 (0)	0 (0)
ApoE e4-positive, *n* (%)					
e3/e4	29 (37.7)	25 (33.8)	32 (42.1)	22 (28.9)	62 (41.6)
e4/e4	5 (6.5)	8 (10.8)	7 (9.2)	11 (14.5)	15 (10.1)
NTB total					
*n*	71	67	73	67	134
Mean (SD)	0.05 (0.66)	− 0.12 (0.61)	0.01 (0.67)	0.05 (0.65)	− 0.03 (0.66)
Median (range)	0.01 (−1.7–1.8)	− 0.09 (− 1.6–1.6)	− 0.03 (− 1.2–1.7)	0.12 (− 1.5–1.9)	0.05 (− 1.7–2.1)
ADAS-Cog11 total					
*n*	70	66	69	69	129
Mean (SD)	16.90 (7.79)	19.23 (7.70)	17.45 (8.05)	19.43 (8.7)	18.52 (8.31)
Median (range)	16.33 (2.3–41.0)	19.00 (4.0–38.0)	15.67 (3.0–39.7)	17.33 (6.0–49.7)	17.00 (2.7–41.7)
CDR-SB total					
*n*	75	73	76	74	148
Mean (SD)	4.75 (2.13)	4.76 (2.23)	4.70 (2.17)	5.18 (2.78)	4.91 (2.43)
Median (range)	4.50 (0.5–12.0)	4.50 (1.0–10.0)	4.50 (0.5–10.0)	5.00 (0.5–14.0)	4.50 (0.5–14.0)

*ADAS-Cog11* Alzheimer’s Disease Assessment Scale-cognitive subscale, *ApoE e4* apolipoprotein E e4 allele, *BID* twice daily, *CDR-SB* Clinical Dementia Rating scale-Sum of Boxes, *NTB* Neuropsychological Test Battery, *QD* once daily, *SD* standard deviation

### Primary endpoint

**Fig. 4:**
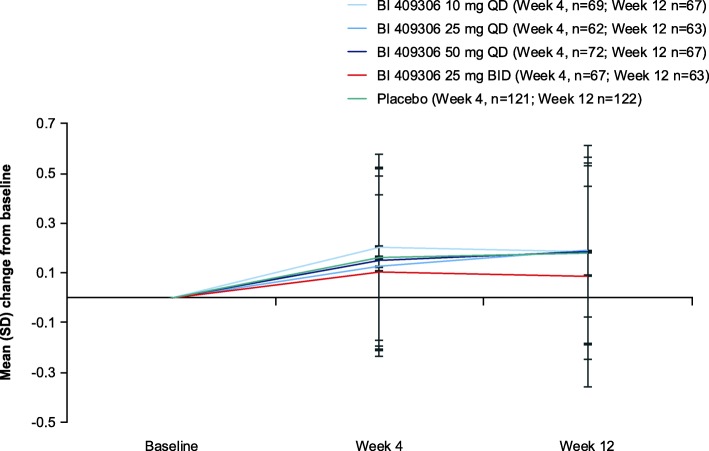
Change from baseline in mean Neuropsychological Test Battery total composite *z*-score by visit for individual BI 409306 doses and placebo (pooled analysis). BID, twice daily; QD, once daily; SD, standard deviation

In the pooled analysis, adjusted mean (standard error [SE]) change from baseline in NTB total composite *z*-score at week 12 was 0.17 (0.03) for pooled BI 409306 doses versus 0.19 (0.04) for placebo. When the scores for both groups were compared, an adjusted mean change of − 0.02 (SE 0.04; 95% confidence interval [CI] − 0.098, 0.061) was observed, which was not statistically significant (*p* = 0.65). Generally, mean NTB total scores showed an increase between baseline and week 4 and remained relatively stable between weeks 4 and 12 (Fig. 4).

Similar results were observed for the individual studies, with an adjusted mean change from baseline of 0.29 (0.03) for BI 409306 pooled doses and 0.27 (0.04) for placebo in study 1, and 0.12 (0.03) and 0.15 (0.05) in study 2, respectively.

The adjusted mean change from baseline in NTB total composite *z*-score at week 12 was also similar across the individual BI 409306 dose groups and placebo in the pooled analysis: BI 409306 10 mg, 0.20 (0.05); 25 mg QD, 0.19 (0.05); 50 mg, 0.19 (0.05); and 25 mg BID, 0.10 (0.05) versus placebo, 0.19 (0.04), and when BI 409306 doses were compared with placebo at week 12, no significant differences were observed (Table 2). Results from the individual studies were consistent with those of the pooled results.

**Table 2:** Summary of change from baseline to week 12 in primary, secondary and further endpoints from the pooled analysis (mixed-model repeated measurement)

Endpoints	BI 409306 10 mg QD (*n* = 67)	BI 409306 25 mg QD (*n* = 63)	BI 409306 50 mg QD (*n* = 67)	BI 409306 25 mg BID (*n* = 63)	Placebo (*n* = 122)
Primary endpoint					
NTB total *z*-score					
Adjusted mean (SE) change from baseline	0.20 (0.046)	0.19 (0.048)	0.19 (0.046)	0.10 (0.047)	0.19 (0.035)
*p* value^*^	0.87	0.95	0.93	0.13	–
Secondary endpoints					
CDR-SB					
Adjusted mean (SE) change from baseline	0.1 (0.170)	0.3 (0.170)	0.1 (0.170)	0.1 (0.170)	0.0 (0.120)
*p* value*	0.82	0.20	0.94	0.66	
ADAS-Cog11					
Adjusted mean (SE) change from baseline	1.13 (0.593)	0.80 (0.623)	0.82 (0.596)	1.32 (0.596)	0.27 (0.444)
*p* value*	0.25	0.49	0.46	0.16	–
Study 1: ADCS-MCI-ADL					
Adjusted mean (SE) change from baseline	0.24 (0.896)	1.79 (0.921)	− 0.10 (0.875)	0.80 (0.947)	0.38 (0.642)
*p* value*	0.90	0.21	0.66	0.72	–
Study 2: ADCS-ADL					
Adjusted mean (SE) change from baseline	0.10 (0.853)	− 0.99 (0.892)	0.35 (0.847)	− 1.07 (0.855)	− 0.58 (0.639)
*p* value*	0.53	0.71	0.38	0.65	–
Further endpoints					
NTB memory domain subscale					
Adjusted mean (SE) change from baseline	0.34 (0.053)	0.27 (0.055)	0.25 (0.053)	0.15 (0.054)	0.26 (0.04)
*p* value*	0.22	0.81	0.92	0.10	–
NTB executive-function domain subscale					
Adjusted mean (SE) change from baseline	− 0.04 (0.062)	− 0.02 (0.064)	0.07 (0.062)	0.01 (0.063)	0.05 (0.046)
*p* value*	0.22	0.38	0.77	0.56	–
NTB immediate-memory domain subscale					
Adjusted mean (SE) change from baseline	0.37 (0.066)	0.32 (0.069)	0.38 (0.067)	0.22 (0.068)	0.38 (0.05)
*p* value*	0.95	0.50	0.95	0.06	–

*ADAS-Cog11* Alzheimer’s Disease Assessment Scale-cognitive subscale, *ADCS-ADL* Alzheimer’s Disease Cooperative Study-Activities of Daily Living scale, *ADCS-MCI-ADL* Alzheimer’s Disease Cooperative Study-Activities of Daily Living for mild cognitive impairment, *BID* twice daily, *CDR-SB* Clinical Dementia Rating scale-Sum of Boxes, *NTB* Neuropsychological Test Battery, *QD* once daily, *SE* standard error

*p values represent the comparison of each dose group with placebo

### Secondary endpoints

#### CDR-SB

In the pooled analysis, adjusted mean (SE) change from baseline in CDR-SB total scores at week 12 was similar between all treatment groups: BI 409306 10 mg, 0.1 (0.17); 25 mg QD, 0.3 (0.17); 50 mg, 0.1 (0.17); 25 mg BID, 0.1 (0.17); and placebo, 0.0 (0.12), and no significant differences in change from baseline in CDR-SB scores were observed between BI 409306 dose groups and placebo (all *p* > 0.05) (Table 2). Similar findings were observed in the two individual studies.

#### ADAS-Cog11

In the pooled analysis, adjusted mean (SE) change from baseline in ADAS-Cog11 total scores at week 12 varied between treatment groups, and no improvements were observed; however, worsening of scores was greatest for the BI 409306 treatment groups: 10 mg, 1.13 (0.59); 25 mg QD, 0.80, (0.62); 50 mg, 0.82 (0.60); and 25 mg BID 1.32 (0.60); compared with the placebo group (0.27 [0.44]). No significant differences were noted when comparing the ADAS-Cog11 total scores for the BI 409306 dose groups with placebo (all *p* > 0.05) (Table 2).

However, in study 1, a comparison of ADAS-Cog11 total scores for BI 409306 dose groups versus placebo showed a significant difference in favour of the 25 mg BID dose: adjusted mean (SE) of − 2.51 (1.07) (95% CI − 4.64, − 0.39; *p* = 0.02). Conversely, in study 2, a significant difference in favour of placebo was observed in the 25 mg BID dose group (adjusted mean [SE] of 2.47 [0.94] [95% CI 0.63, 4.31; *p* = 0.009]). No significant differences were reported for the other BI 409306 dose groups in either study 1 or 2.

#### ADCS-MCI-ADL and ADCS-ADL

In study 1, adjusted mean (SE) change from baseline in ADCS-MCI-ADL total score at week 12 varied between treatment groups, with the greatest improvement observed with the BI 409306 25 mg dose: BI 409306 10 mg, 0.24 (0.90); 25 mg QD, 1.79 (0.92); 50 mg, − 0.10 (0.88); 25 mg BID, 0.80 (0.95); and placebo, 0.38 (0.64). When the ADCS-MCI-ADL total scores for BI 409306 dose groups were compared with placebo, no significant differences in adjusted mean (SE) scores were observed: 10 mg, − 0.14 (1.10); 25 mg QD, 1.40 (1.12); 50 mg, − 0.49 (1.09); and 25 mg BID, 0.42 (1.14) (Table 2).

In study 2, adjusted mean (SE) change from baseline in ADCS-ADL total score at week 12 also varied between study groups; however, in this cohort of patients with mild AD, the greatest improvement was observed with the BI 409306 50 mg dose: BI 409306 10 mg, 0.10 (0.85); 25 mg QD, − 0.99 (0.89); 50 mg, 0.35 (0.85); 25 mg BID, − 1.07 (0.86); and placebo, − 0.58 (0.64). When compared with placebo, no significant differences were observed for any BI 409306 doses (all *p* > 0.05) (Table 2).

### Additional endpoint

#### NTB subscale responses

In the pooled analysis, adjusted mean (SE) change from baseline in NTB memory domain *z*-score at week 12 was similar across all BI 409306 doses and placebo, with the greatest improvement observed for the BI 409306 10 mg dose: 0.34 (0.05) versus 25 mg QD, 0.27 (0.06); 50 mg, 0.25 (0.05); 25 mg BID, 0.15 (0.05); and placebo, 0.26 (0.04). There were no significant differences between any BI 409306 doses and placebo (all *p* > 0.05) (Table 2), and similar findings were reported with the individual studies.

In the pooled analysis, adjusted mean (SE) change from baseline in NTB executive-function domain *z*-score at week 12 was similar across all treatment groups: BI 409306 10 mg, − 0.04 (0.06); 25 mg QD, − 0.02 (0.06); 50 mg, 0.07 (0.06); 25 mg BID, 0.01 (0.06); and placebo, 0.05 (0.05). When BI 409306 dose groups were compared with placebo, no significant differences were observed in adjusted mean scores for this domain (all *p* > 0.05) (Table 2). These findings were similar across the individual studies, except in study 1, where a significant difference was observed in NTB executive-function domain *z*-score at week 12 in favour of the BI 409306 25 mg BID dose, compared with placebo (adjusted mean [SE] of 0.28 [0.11] [95% CI 0.060, 0.506; *p* = 0.013]). No significant differences were reported among the other BI 409306 dose groups in either study.

For NTB immediate-memory domain *z*-scores at week 12 in the pooled analysis, the adjusted mean (SE) change from baseline was similar across all treatment groups: BI 409306 10 mg: 0.37 (0.07); 25 mg QD, 0.32 (0.07); 50 mg, 0.38 (0.07); 25 mg BID, 0.22 (0.07); and placebo, 0.38 (0.05). No significant differences were observed in adjusted mean (SE) scores across BI 409306 doses versus placebo for this domain (Table 2). Similarly, no significant differences between treatment groups were observed within the individual studies.

### Safety analyses

In general, BI 409306 was well tolerated. The highest frequencies of AEs and drug-related AEs occurred in the BI 409306 50 mg dose group (59.2% and 19.7%, respectively; Table 3), and the percentages of SAEs were highest in the BI 409306 25 mg BID dose and placebo groups (5.3% and 6.0%, respectively; Table 3). All SAEs experienced by patients receiving BI 409306 25 mg BID required, or prolonged, hospitalisation. One patient in the BI 409306 10 mg dose group experienced an AE leading to death (Alzheimer’s-type dementia and encephalopathy), but this was not considered related to the study drug. In total, 10 patients (2.2% of the pooled treated set) discontinued treatment due to AEs (*n* = 5 across all BI 409306 doses and *n* = 5 receiving placebo). There were no clinically significant differences between treatment groups in laboratory parameters, physical and neurological examinations, or vital signs. AEs of suicidal ideation without intent occurred in 2 patients receiving BI 409306 (25 mg BID) and 1 patient receiving placebo. Safety findings for the individual studies were consistent with those from the pooled analysis.

**Table 3:** Summary of reported AEs from the pooled analysis

n (%)	BI 409306 10 mg QD (*n* = 77)	BI 409306 25 mg QD (*n* = 74)	BI 409306 50 mg QD (*n* = 76)	BI 409306 25 mg BID (*n* = 76)	Placebo (*n* = 149)
Patients with any AE	34 (44.2)	30 (40.5)	45 (59.2)	37 (48.7)	67 (45.0)
Patients with severe AEs*	2 (2.6)	1 (1.4)	1 (1.3)	1 (1.3)	1 (0.7)
Patients with investigator-defined, drug-related AEs	5 (6.5)	5 (6.8)	15 (19.7)	8 (10.5)	12 (8.1)
Patients with AEs leading to discontinuation of trial drug	1 (1.3)	1 (1.4)	2 (2.6)	1 (1.3)	5 (3.4)
Patients with AEs of special interest	0 (0)	0 (0)	0 (0)	0 (0)	0 (0)
Patients with SAEs	1 (1.3)	3 (4.1)	1 (1.3)	4 (5.3)	9 (6.0)
Resulted in death	1 (1.3)	0 (0)	0 (0)	0 (0)	0 (0)
Was life-threatening	0 (0)	0 (0)	1 (1.3)	0 (0)	0 (0)
Persisted or caused significant disability/incapacity	0 (0)	0 (0)	0 (0)	0 (0)	0 (0)
Required, or prolonged, hospitalisation	1 (1.3)	2 (2.7)	0 (0)	4 (5.3)	7 (4.7)
Congenital anomaly or birth defect	0 (0)	0 (0)	0 (0)	0 (0)	0 (0)
Other medically important serious event	0 (0)	1 (1.4)	0 (0)	1 (1.3)	2 (1.3)
Patients with other significant AEs (according to ICH E3)	0 (0)	0 (0)	1 (1.3)	0 (0)	4 (2.7)

*There was one subject with AEs leading to death (BI 409306 10 mg group). *AE* adverse event, *BID* twice daily, *ICH E3* International Conference on Harmonisation of Technical Requirements for Registration of Pharmaceuticals for Human Use, Guideline E3, *QD* once daily, *SAE* serious adverse event

## Discussion

This pooled analysis of two phase II proof-of-concept studies is the first evaluation of the efficacy and safety of a PDE9 inhibitor in prodromal and mild AD. BI 409306 was administered at doses of 10–50 mg daily over a 12-week treatment period.

BI 409306 was well tolerated, and there were no concerns with respect to safety. The highest frequency of AEs was reported in the BI 409306 50 mg dose group, and the incidence of SAEs in drug-treated patients was lower than that seen with placebo. Safety findings for the individual studies were in keeping with those from the pooled analysis.

The pooled analysis of the primary endpoint revealed no significant changes in the NTB total composite *z*-score at week 12 in the BI 409306 treatment groups compared with placebo, with similar findings observed in the individual studies. The analysis of all secondary endpoints, including a pooled analysis of CDR-SB and ADAS-Cog11, and ADCS-MCI-ADL (for study 1), ADCS-ADL (for study 2), also gave no indication of a treatment benefit for BI 409306. Similarly, findings from the pooled analysis of the additional endpoint (NTB subscale scores) revealed no significant differences between BI 409306 (all dose groups) and placebo. Overall, these data do not demonstrate efficacy of BI 409306 in improving cognition in patients with prodromal and mild AD.

A previous 12-week, phase II clinical trial assessing the efficacy and safety of a different PDE9 inhibitor, PF-04447943 25 mg BID, in patients with mild-to-moderate probable AD produced similar results versus placebo as the present study; the drug was generally well tolerated, but failed to improve cognition and behaviour, compared with placebo [19]. As both studies were of 12 weeks’ duration, it may be that this time period is too short to reveal any statistically and clinically significant effects of treatment, limiting interpretability. However, given the lack of efficacy on a range of endpoints after 12 weeks of treatment and 4 weeks of follow-up, it seems unlikely that continued treatment with BI 409306 would have produced significant improvements in this study. One potential explanation for the lack of efficacy could be that the effects of PDE9 inhibition may be minimised in the presence of severe or diffuse lesions, as are often present in clinical AD, even in the prodromal stage of the disease [20]. Moreover, finding the optimum effective dose of a PDE inhibitor has proved to be difficult [21], and the doses chosen for evaluation may not have been appropriate (although the inclusion of 4 separate BI 409306 doses in the present study increased the chance of success at using the optimum dose, in contrast to the prior study, which only tested a single dose of PF-04447943 of 25 mg BID).

The change from baseline for the NTB total score was chosen as the primary endpoint for the present studies, instead of the more commonly used ADAS-Cog. This selection was based primarily on evidence that the NTB may be a more sensitive and appropriate measure of cognition in the early stages of AD than ADAS-Cog [22]. Importantly, the NTB includes tests of both memory and executive function, whereas the ADAS-Cog does not include measures of the latter [18, 22]. While moderate practice effects on the NTB, but not the ADAS-Cog, total score were observed in the present studies, results for both instruments were similar in showing no apparent benefit over placebo. The practice effects on NTB are well known and were observed on memory, but not executive function, items of the NTB.

Both studies originally planned to randomise similar numbers of patients. However, while the target for recruitment was met in study 2, fewer patients were recruited for study 1. This was due to the limited availability of patients with prodromal AD and the high screening failure rate among these patients, which are common challenges in studies of prodromal AD. However, there was an increase in the number of patients in each treatment group following the removal of the donepezil arm from the study design that ensured adequate power for the planned analyses, including the combined analysis.

The criteria used for patient selection varied somewhat between the two studies. The diagnosis of prodromal AD in study 1 was based upon the IWG criteria [14], whereas the diagnosis of probable mild AD in study 2 was based on NIA-AA criteria [15]. Therefore, while the diagnosis of mild AD was based primarily on clinical symptomatology, namely the presence of core clinical criteria for dementia along with additional differentiating criteria, the diagnosis of prodromal AD was based on the presence of subjective and objective MCI, along with positive findings of amyloid biomarkers of AD. The lack of requirement for positive biomarker evidence of AD is often cited as a concern in AD dementia studies, as some patients may meet criteria for the clinical diagnosis of probable AD who in fact do not have AD. While this is a genuine methodological concern, similar results were seen across the two studies, suggesting that lack of biomarker requirements was not likely a primary cause of the negative results overall. Additionally, 45% of patients in study 2 were carriers of the apolipoprotein E e4 allele and results in this subgroup were not notably different from the overall results.

## Conclusions

In patients with prodromal or mild AD, no clinically meaningful changes in scores for NTB (total and subscale scores), CDR-SB, ADAS-Cog11, and ADCS-MCI-ADL and ADCS-ADL were observed across a range of BI 409306 doses. BI 409306 was generally well tolerated in this patient population.

## Abbreviations

AChEI: Acetylcholinesterase inhibitor
AD: Alzheimer’s disease
ADAS-Cog11: Alzheimer’s Disease Assessment Scale-cognitive subscale
ADCS-ADL: Alzheimer’s Disease Cooperative Study-Activities of Daily Living scale
ADCS-MCI-ADL: Alzheimer’s Disease Cooperative Study-Activities of Daily Living scale for mild cognitive impairment
AE: Adverse event
BID: Twice daily
CDR: Clinical Dementia Rating scale
CDR-SB: Clinical Dementia Rating scale-Sum of Boxes
cGMP: Cyclic guanosine monophosphate
CI: Confidence interval
CSF: Cerebrospinal fluid
GCP: Guideline for Good Clinical Practice
ICH: International Council for Harmonisation
LTP: Long-term potentiation
MCI: Mild cognitive impairment
MMSE: Mini-Mental State Examination
NMDA: *N*-Methyl-d-aspartate
NTB: Neuropsychological Test Battery
PDE9: Phosphodiesterase type 9
QD: Once daily
REML: Restricted maximum likelihood
SAE: Serious adverse event
SD: Standard deviation
SE: Standard error

## References

[1] Alzheimer's Association (2016). 2016 Alzheimer’s disease facts and figures. Alzheimers Dement.

[2] Fiest KM (2016). The prevalence and incidence of dementia due to Alzheimer’s disease: a systematic review and meta-analysis. Can J Neurol Sci.

[3] Dubois B (2010). Revising the definition of Alzheimer’s disease: a new lexicon. Lancet Neurol.

[4] Lee HG (2002). Differential regulation of glutamate receptors in Alzheimer’s disease. Neurosignals.

[5] Bales KR (2010). Phosphodiesterase inhibition to target the synaptic dysfunction in Alzheimer’s disease. Topics Med Chem.

[6] Reneerkens OA (2009). Selective phosphodiesterase inhibitors: a promising target for cognition enhancement. Psychopharmacology.

[7] Moschetti V (2016). First-in-human study assessing safety, tolerability and pharmacokinetics of BI 409306, a selective phosphodiesterase 9A inhibitor, in healthy males. Br J Clin Pharmacol.

[8] Butterfield DA, Pocernich CB (2003). The glutamatergic system and Alzheimer’s disease: therapeutic implications. CNS Drugs.

[9] Ricciarelli R, Fedele E (2018). cAMP, cGMP and amyloid β: three ideal partners for memory formation. Trends Neurosci.

[10] Rosenbrock H (2015). BI 409306, a novel phosphodiesterase 9A inhibitor, part II: in-vivo characterization regarding target engagement and cognition tasks in rodents. Schizophr Bull.

[11] Rosenbrock H (2015). Improving synaptic plasticity and cognitive function in rodents by the novel phosphodiesterase 9A inhibitor BI 409306. Alzheimers Dementia.

[12] Rosenbrock H (2015). Pharmacological characterization of the novel phosphodiesterase 9A inhibitor BI 409306 in animal models related to synaptic plasticity and cognitive function. Biol Psychiatry.

[13] Dorner-Ciossek C, Giovannini R, Rosenbrock H (2015). BI 409306, a novel phosphodiesterase 9A inhibitor, part I: potency, selectivity and in-vitro functional characterization on synaptic plasticity. Schizophr Bull.

[14] Dubois B (2014). Advancing research diagnostic criteria for Alzheimer’s disease: the IWG-2 criteria. Lancet Neurol.

[15] Hyman BT (2012). National Institute on Aging–Alzheimer’s Association guidelines for the neuropathologic assessment of Alzheimer's disease. Alzheimers Dementia.

[16] International Conference on Harmonisation of Technical Requirements for Registration of Pharmaceuticals for Human Use: Guideline for Good Clinical Practice E6 (1). 1996.

[17] World Medical Association (2013). Declaration of Helsinki - ethical principles for medical research involving human subjects.

[18] Harrison J (2007). A neuropsychological test battery for use in Alzheimer disease clinical trials. Arch Neurol.

[19] Schwam EM (2014). A multicenter, double-blind, placebo-controlled trial of the PDE9A inhibitor, PF-04447943, in Alzheimer’s disease. Curr Alzheimer Res.

[20] Aisen PS (2017). On the path to 2025: understanding the Alzheimer’s disease continuum. Alzheimers Res Ther.

[21] Prickaerts J, Heckman PRA, Blokland A (2017). Investigational phosphodiesterase inhibitors in phase I and phase II clinical trials for Alzheimer’s disease. Expert Opin Investig Drugs.

[22] Cummings JL (2008). Controversies in Alzheimer’s disease drug development. Int Rev Psychiatry.

